# Dialectical Core Schemas Mediate the Relationships Between Dissociative Experiences and Symptomatology in a Community Sample

**DOI:** 10.1177/00332941231175065

**Published:** 2023-05-22

**Authors:** Bruno Faustino, Pamela Pilkington, Patrícia M. Pascoal

**Affiliations:** Lusófona University, HEI‐Lab: Digital Human‐Environment Interaction Labs, Portugal; Faculdade de Psicologia da Universidade de Lisboa, Lisboa, Portugal; Australian Catholic University, Fitzroy, VI, Australia; Lusófona University, HEI‐Lab: Digital Human‐Environment Interaction Labs, Portugal

**Keywords:** Dialectical core schemas, dissociative experiences, symptomatology

## Abstract

**Background:**

Maladaptive cognitions about the self and others are associated with a wide array of dysfunctional responses and psychopathological symptoms in non-clinical and clinical samples. Dissociative experiences (e.g., depersonalization and derealization) as a coping response to stressful situations lie on a continuum from healthy to unhealthy but are generally elevated in individuals experiencing mental illness. However, the extent to which Dialectical Core Schemas explain the relationship between dissociative experiences and symptomatology is unclear. Therefore, this study aimed to investigate the mediating role of Dialectical Core Schemas on the relationship between dissociative experiences and symptomatology.

**Methods:**

A community sample of 179 participants were recruited (*M*_*age*_ = 21.2 years, *SD* = 8.2). Data were gathered through self-report questionnaires using a cross-sectional design.

**Results:**

Maladaptive core schemas about self and others correlated positively with all dissociative experiences (e.g., depersonalization/derealization, amnesia), while adaptive core schemas about the self correlated negatively with depersonalization/derealization and distractibility. Maladaptive core schemas mediated the relationship between dissociative experiences and symptomatology.

**Conclusions:**

The relationships between dissociative experiences and symptomatology are bi-directional. Exploring the mediating factors may help clinicians and researchers better understand how to enhance case conceptualization and clinical decision-making.

## Introduction

### Dialectical Core Schemas as an Alternative Schema Perspective

According to cognitive therapy models, schemas are extreme beliefs about oneself, others, the world, and the future, that increase one’s vulnerability to psychopathology (Beck et al., 2015; Young, et al., 2003). These mental representations, referred to as maladaptive schemas, are rigid, extreme, and trait-like and tend to guide cognition, emotion, and behavior. Several schema concepts have been described in clinical literature, such as cognitive schemas (Beck et al., 1979), early maladaptive schemas (EMS, Young, et al., 2003), emotional schemas (Leahy, 2002), interpersonal schemas (Hill & Saffran, 1995), pathogenic beliefs (Aafjes van Doorn et al., 2021), and self-wounds (Wolfe, 2005). The shared tendency/commonality between these concepts is the focus on maladaptive schemas that originated in early experiences. However, recent research has suggested that positive schemas have empirical support and may be an asset in case conceptualization (Louis et al., 2018; Paetsch et al., 2022). Therefore, a different and integrative concept of dialectical core schemas was suggested.

Dialectical core schemas (DCS) are psychological structures with maladaptive and adaptive core representations about the self and others resulting from the adverse and/or adaptive experiences that individuals experience in their lifetime (Faustino, 2022a, 2022b). Self and other cognitions are organized on dialectical poles lying on a continuum from maladaptive and adaptive. Specifically, these self-other representations emerge from the dialectical negotiation between the experiential and reflexive self, which underlie the meaning-making processes (Faustino, 2022a, 2022b). Therefore, DCS are organized into four domains articulated dialectically on a continuum: (1) maladaptive self, (2) adaptive self, (3) maladaptive other, and (4) adaptive other.

The Brief Core Schemas Scale (BCSS, Fowler et al., 2006) can be used to operationalize the DCS construct because it resembles the same theoretical structure, with positive and negative schemas about the self and others. Negative schemas measured with the BCSS tend to be associated with generic psychopathological symptoms (Faustino, 2022a) and paranoia (Fowler et al., 2006). Nevertheless, research on the BCSS with other psychopathological-related variables is sparse. Previous research suggests dysfunctional core beliefs or maladaptive schemas are associated with various psychiatric disorders and psychological constructs. EMS measured by the YSQ-S3 (Young et al., 2005) are associated with eating disorders (Maher et al., 2022), affective disorders (Nilsson, 2016), depressive disorders (Bishop et al., 2022), anxiety disorders (Tariq et al., 2021), post-traumatic stress disorder (Cockram et al., 2010), personality disorders (Barazandeh et al., 2016; Kunst et al., 2020) and symptomatology in non-clinical samples (Carr & Francis, 2010). Dysfunctional core beliefs measured by the PBQ-SQ (Butler et al., 2007) are also associated with personality disorders (Beck et al., 2001) and symptomatology in non-clinical samples (Faustino et al., 2022a). Also, EMS is related to interpersonal dysfunctional cycles (Faustino & Vasco, 2020c), cognitive fusion and psychological needs (Faustino & Vasco, 2020a), emotional processing difficulties (Faustino & Vasco, 2020b), defense mechanisms (Walburg & Chiaramello, 2015), and dissociative experiences (Bortolon et al., 2017). If the results of the present study are in line with previous research, it can be suggested that the BCSS may be a valid instrument to identify the schematic functioning and, eventually, will be able to operationalize the DCS construct.

Moreover, the contribution of maladaptive schemas to emotional trauma and distress outcomes may be twofold: EMS can be regarded as risk factors, perpetuating factors, and mediators. For example, Roelofs and colleagues (2011) showed that EMS mediated the quality of attachment relationships and depressive symptomatology; Vasilopoulou and colleagues (2020) showed that EMS mediates the relationship between childhood traumatic events and complex post-traumatic stress disorder. Similarly, Khosravi (2020), showed that EMS of vulnerability to harm and defectiveness/shame were significant mediators of the relationships between emotional neglect and dissociation. Faustino & Vasco, 2020a showed that EMS mediated the relationships between emotional processing difficulties and symptomatology; Costa et al. (2022) described how EMS mediated the relationship between early life stress and psychiatric symptoms; and finally, Faustino et al., 2022b showed that the EMS of vulnerability to harm and mistrust abuse mediated the negative relationship between COVID-19 anxiety and life satisfaction. This empirical evidence supports the role of EMS as a cognitive mediator of the relationships between various risk factors and mental health and well-being outcomes.

However, despite this evidence on EMS, it is not clear if DCS have a mediating role in the relationship between dissociative experiences and symptomatology in a non-clinical sample. Thus, the DCS construct is somewhat similar but also different from the EMS construct because: (1) it is focused on core self-other cognitive representations, (2) it does not depend exclusively on early experiences, (3) it has a dimensional structure—adaptive and maladaptive, and (4) it has two domains which can be independent (self and other). Therefore, the current study sought to explore how DCS relate to dissociative experiences.

### Dissociation and Dissociative Experiences

Dissociation may be viewed as a response and/or a defense mechanism (Cramer, 2000) to life events that enables the individual to disconnect from or minimize awareness of distressing thoughts, sensations, feelings, perceptions, bodily sensations, sense of self, behaviors, or memories (Waller et al., 1996). Dissociative experiences result from the dissociation process in every day, transient, peri-traumatic, and/or maladaptive (Waller et al., 1996). According to Seligman and Kirmayer (2008), dissociative experiences have been conceptualized as dimensional, lying on a continuum from everyday experiences, such as becoming absorbed by one thought or emotion to depersonalized due to trauma. The intensity of dissociated experiences relies on the type of experience; in this sense, dissociative experiences may be recurrent in non-clinical populations in a not pathological manner (Seligman & Kirmayer, 2008). Thus, the Diagnostic and Statistical Manual of Mental Disorders, fifth edition (DSM-5, pp. 291), defines dissociation “as a disruption, interruption, and/or discontinuity of the normal, subjective integration of behavior, memory, identity, consciousness, emotion, perception, body representation, and motor control”. DSM-5 diagnostic criteria also include dissociative disorders (e.g., dissociative identity disorder, DID), (complex) post-traumatic stress disorder (CPTSD, PTSD), and borderline personality disorder (BPD).

Typically, four types of dissociative experiences are described, such as Depersonalization-derealization (associated with experiences of feeling disconnected, separated from oneself, one’s body, and other people); Absorption (related to experiences of involvement in an activity, memory, or fantasy with detachment from the environment, the ability to ignore the pain and being able to do usually difficult things); Distractibility (includes experiences of losing part of the information of events, of finding things that one does not remember to do and confusion about memories) and Amnesia (involves severe forgetfulness) (Bernstein & Putnam, 1986). These experiences tend to be assessed with the Dissociative Experiences Scale (DES-28, Bernstein & Putnam, 1986). Previous research showed that dissociative experiences are associated with post-traumatic stress disorder (PTSD, Waelde et al., 2009), depression (Fung et al., 2020; Parlar et al., 2017), psychosis (Laddis & Dell, 2012), borderline personality disorder, and trauma (Krause-Utz, 2022). A recent meta-analysis by Lyssenko et al. (2018) of 216 studies showed that dissociative experiences (measured with the DES-28), were prevalent in several psychiatric disorders, such as dissociative disorders, posttraumatic stress disorder, borderline personality disorder, and conversion disorder. If dissociation and dissociative experiences are present in several psychopathological disorders, they may represent transdiagnostic coping mechanisms activated by distressing experiences (Ellickson-Larew et al., 2020; Ociskova et al., 2015). Recent studies suggested that individuals experience a wide array of symptoms rather than discrete singular categorial entities (Dalgleish et al., 2020). According to the transdiagnostic perspective of psychopathology, latent factors are responsible for the shared mechanisms that articulate several psychopathological symptoms (Kotov et al., 2017). Several variables are being purposed as core transdiagnostic variables (Lynch et al., 2021) which supports the need to explore the DCS construct and dissociative experiences within a transdiagnostic perspective with generic symptomatology. Although the connection between dissociative experiences and psychopathological symptoms is well established, the interrelationships between dissociation, DCS, and psychological symptoms are yet to be explored.

### The Current Study

We theorized that the relationships between dissociative experiences and symptomatology are mediated through the core self-schemas, where the maladaptive core self has a facilitating role, while the adaptive core self has an inverse role. The same can be said for the maladaptive and adaptive other schemas. Dissociative mechanisms range from adaptive to maladaptive, and if individuals experience psychological distress, some dissociative mechanisms may likely be used. If these coping mechanisms are severe, psychopathological symptoms may occur. However, if individuals see themselves as flawed, unworthy, weak, and/or undeserving of affect and see others as deceitful, bad, harsh, and/or unforgiving then it is expected that the relationship between dissociative experiences and psychopathological symptoms would be enhanced. The same can be said for adaptive views of the self and others, which will weaken this relationship. As stated previously, maladaptive schemas are significant mediators of several relationships (e.g., childhood traumatic events and CPSDS); however, the relationships with DCS are still unexplored. Therefore, the following hypotheses were formed:

H1 Maladaptive core schemas are positively associated with dissociative experiences;

H2 Adaptive core schemas are negatively associated with dissociative experiences;

H3 Maladaptive schemas and dissociative experiences predict symptomatology;

H4 Maladaptive and Adaptive core-self schemas mediate the relationship between dissociative experiences and symptomatology;

H5 Maladaptive and Adaptive core-other schemas mediate the relationship between dissociative experiences and symptomatology.

## Method

### Participants and Procedures

Participants were recruited from the undergraduate course of psychology through the Qualtrics platform. All participants gave informed consent in the online platform to participate in the present study. Inclusion criteria were being aged 18–65 years, speaking Portuguese for more than 5 years, and not having a self-reported neurocognitive disorder. This research was approved by the ethics committee of BLINDED. The sample consisted of 179 participants (86% female). The age of the men varied between 18 and 65 years (M = 28.2, *SD* = 15.1), and the age of the women varied between 18 and 59 years (M = 20.1, *SD* = 5.78). Participants’ nationalities were primarily Portuguese (*n* = 167; 93.3%). All participants were Portuguese speakers with more than 5 years of proficiency. Most participants’ highest level of education was 12th grade (*n* = 153; 85.5%), while 18 (10.1%) had a Undergraduate degree, and 8 (4.5%) had a Master’s or Doctoral degree. Marital status was the following: 170 were single (95%), seven were married (3.9%), one was co-habitant (.6%), and one was divorced (.6%).

## Ethical Statement

The Declaration of Helsinki research principles were used in the present study, and it was approved by the Ethical Committee of the University of Lisbon. We declare the following aspects of the present study: (1) all participants gave informed consent to participate in the study; (2) the scientific purpose of the research was explained; (3) privacy and anonymity were made clear in the instructions; (4) this paper reflects an original unpublished scientific study; (5) authors of the study declare that there is no conflict of interest and (6) data is not shared, because it belongs to an ongoing project.

### Measures

#### Brief Core Schemas Scales (BCSS)

The BCSS (Fowler, et al., 2006, translated and adapted to European Portuguese by Faustino, 2022a) was used to assess adaptive and maladaptive core schemas about the self and others. It is a self-report questionnaire with 24 items divided into four dimensions with a five-point rating scale (0–4). The adaptive self-subscale contains items such as “I am interesting”, and “I am talented”; the maladaptive self-subscale contains items such as “I am worthless”, and “I am vulnerable”; The adaptive others-subscale contains items such as “Other people are fair” and “Other people are good” and the maladaptive others-subscale contains items such as “Other people are hostile”, “Other people are harsh”. Subscale scores range from 0 to 20, with higher scores indicating higher schema endorsement. Cronbach’s alpha of the four subscales was as follows: adaptive core self-schemas (α = .78), maladaptive core self-schemas (α = .79), adaptive core other-schemas (α = .85), and maladaptive core other-schemas (α = .81).

#### Dissociative Experience Scales (DES-28)

The DES-28 (Bernstein & Putnam, 1986, translated and adapted to European Portuguese by Espirito Santo & Pio Abreu, 2008) self-response instrument that aims to assess four distinct dissociative experiences: depersonalization/derealization, absorption, distractibility, and amnesia. It has 28 items answered on a Likert scale from 0 to 100. Items are summed to form a total score, and four subscales represent the four types of dissociative experiences. Higher scores indicate greater dissociation. Cronbach’s alpha of the total score was (α = .97), depersonalization/derealization (α = .83), absorption (α = .82), distractibility (α = .79), and amnesia (α = .71).

#### Brief Symptoms Inventory (BSI-53)

The BSI-53 (Derogatis, 1993, translated and adapted to European Portuguese by Canavarro, 1999) assessed psychopathological symptoms, including anxiety, psychoticism, depression, or interpersonal sensibility. BSI-53 is a self-report questionnaire with 53 items on a five-point Likert scale (0–4). Only the total index was used, and Cronbach’s alpha for the total score was high (α = .97).

#### Statistical Analyses

Normal distribution was tested through the analysis of Skewness and Kurtosis. Values were between −2 to +2 and between −7 to +7, respectively, suggesting a normal data distribution (Byrne, 2010)—see Table 1. Multicollinearity was analyzed and proved adequate for all analyses |VIF < 5; T < 2| (Pallant, 2007). The sample was described with descriptive statistics. Pearson correlations were used to explore associations between schemas, dissociative experiences, and symptoms. A hierarchical regression with stepwise criteria was used to explore the additive predictive value of schemas and dissociative experiences on symptomatology. Mediation analyses were performed using the PROCESS macro for SPSS (Hayes, 2013). Confidence intervals of 95% and 10,000 bootstrap computations were used with a significance level of *p* < .05. G power was used to conduct a power analysis for the mediation models. A sample size of 119 was required for a .95 of actual power (α = .05, f2 = .15). The study sample is larger (*N* = 179) than required for the analysis. All analyses were performed in the SPSS version 25.

**Table 1.** Descriptive Statistics and Normality Indexes for Variables Under Study (*N* = 176).

	*M*	*SD*	Min	Max	SK	K
Maladaptive core self schemas (BCSS)	1.67	.58	1.00	3.83	1.30	1.60
Adaptive core self schemas (BCSS)	2.55	.58	1.00	4.00	−.13	−.45
Maladaptive core other schemas (BCSS)	1.70	.47	1.00	3.33	.51	−.08
Adaptive core other schemas (BCSS)	2.19	.51	1.00	4.00	.48	1.00
DES-28 total score	1.37	.58	.14	3.03	−.01	.14
Depersonalization/derealization (DES-28)	1.31	1.20	.00	9.86	2.0	4.60
Absorption (DES-28)	3.24	2.10	.00	8.71	.39	−.76
Distractibility (DES-28)	21.62	12.37	1.00	59.43	.56	−.24
Amnesia (DES-28)	4.10	5.49	.00	32.33	2.20	5.80
Symptomatology (BSI-53)	1.21	.75	.13	3.40	−.07	.01

Note: BCSS = brief core schema scales; DES-28 = dissociative experiences scale; BSI-53 = brief symptoms inventory.

## Results

Table 1 provides the means, standard deviations, minimum, maximum, skewness, and kurtosis of the maladaptive and adaptive self-other schemas (BCSS), dissociative experiences (DES-28), and symptomatology (BSI-53).

Table 2 provides the Pearson correlations between the variables. Maladaptive core self-schemas correlated positively with DES-28 Total Score (r = .39, *p* < .05), depersonalization/derealization (r = .48, *p* < .05), absorption (r = .33 *p* < .05), distractibility (r = .25, *p* < .05), amnesia (r = .21, *p* < .05), and symptomatology (r = .62, *p* < .05). Adaptive core self-schemas only correlated negatively with DES-28 Total Score (r = −.23, *p* < .05), depersonalization/derealization (r = −.34, *p* < .05), distractibility (r = −.19, *p* < .05), and symptomatology (r = −.50, *p* < .05). Maladaptive core other-schemas showed a similar pattern with maladaptive core self-schemas (*p* < .05) and adaptive core other-schemas did not correlate with any variable (see Table 2).

**Table 2.** Pearson Correlations Between Dialectical Core Schemas, Dissociation, and Symptomatology (*N* = 179).

	DES-28 Total Score	DEP/DES	ABS	DIS	AMNS	BSI-53
Maladaptive core self-schemas	.39^**^	.48^**^	.33^**^	.25^**^	.21^**^	.62^**^
Adaptive core self-schemas	−.23^**^	−.34^**^	−.13	−.19^*^	−.11	−.50^**^
Maladaptive core other-schemas	.24^**^	.22^**^	.22^**^	.25^**^	.14	.34^**^
Adaptive core other-schemas	−.08	−.10	−.03	−.12	−.05	−.11
Symptomatology (BSI-53)	.57^**^	.55^**^	.48^**^	.48^**^	.39^**^	—

Note: **p < .01; *p < .05.

DEP/DES = depersonalization/derealization (DES-28); ABS = absorption (DES-28); DIS = distractibility (DES-28); AMNS = amnesia (DES-28); BSI-53 = symptomatology (BSI-53).

Table 3 describes a hierarchical regression analysis with stepwise criterion with dialectical core schemas and dissociative experiences as independent variables and with symptomatology as a dependent variable. A statistically significant model with five predictors was found, with maladaptive self and other schemas, adaptive self-schemas, distractibility, and amnesia, which accounts for 55% (b = .15, *p* < .01) of the variance of symptomatology in the present sample (see Table 3). The stepwise criteria remove the nonsignificant variables from the analysis. All variables from the BCSS and DES-28 were used. A significant model is shown in Table 3. Removed variables were the following: Adaptive core self-schemas (BCSS), depersonalization/derealization and absorption from DES-28.

**Table 3.** Hierarchical Regression Analysis With Dialectical Core Schemas and Dissociative Experiences as Independent Variables and With Symptomatology as a Dependent Variable (*N* = 179).

	*R* ^*2*^	*B*	Error	*b*	*t*	Sig
Maladaptive core self schemas	.38	.51	.08	.40	6.428	.01
Maladaptive core other schemas	.43	.24	.08	.15	2.881	.04
Adaptive core self schemas	.45	−.24	.08	−.19	−3.127	.02
Distractibility (DES-28)	.54	.01	.04	.23	3.897	.01
Amnesia (DES-28)	.55	.02	.08	.15	2.539	.01

**Figure 1.**
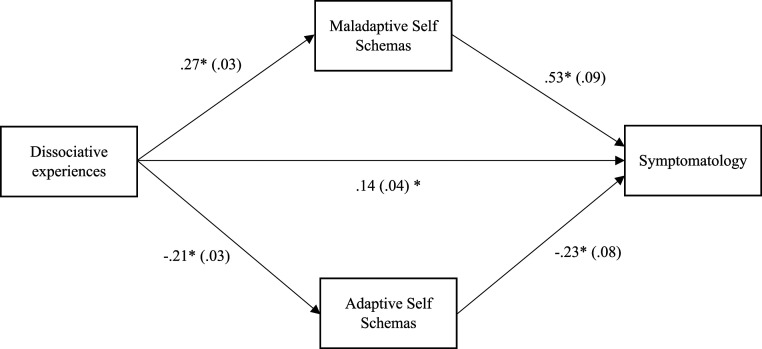
Mediation analysis of the relationship between dissociation and symptomatology with maladaptive and adaptive core self-schemas as mediators.

Two mediation models were tested. The first model was statistically significant. The indirect effect between dissociative experiences and symptomatology was significant (b = .14, |.04 −.22|, *p* < .05). The relationship between dissociative experiences and symptomatology was mediated by maladaptive core self-schemas (ab = .14, |.08–.21|, *p* < .05). The relationship between dissociative experiences and symptomatology was also mediated by adaptive core self-schemas (ab = .05, |.01–.09|, *p* < .05). See Figure 1.

**Figure 2.**
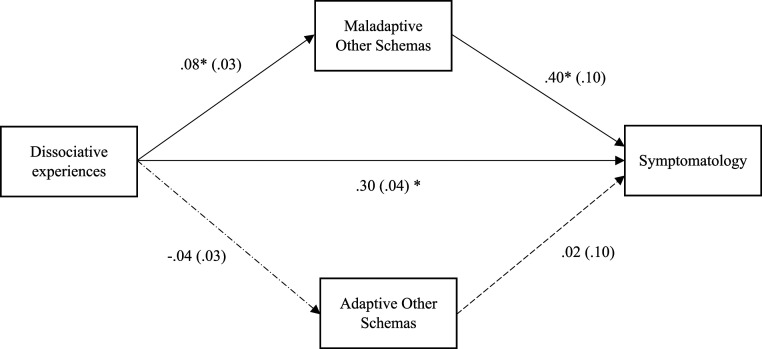
Mediation analysis of the relationship between dissociation and symptomatology with maladaptive and adaptive core other schemas as mediators.

The second model was partially statistically significant. The indirect effect between dissociative experiences and symptomatology was significant (b = .30 |.21–.38|, *p* < .05). The relationship between dissociative experiences and symptomatology was mediated by maladaptive other schemas (ab = 03, |.01–.07|, *p* < .05). However, the relationship between dissociative experiences and symptomatology was not mediated by adaptive core other schemas. See Figure 2.

## Discussion

This study aimed to explore the role of Dialectical Core Schemas in the relationship between Dissociative Experiences and symptomatology. Results were partially in line with theoretical predictions. Hypothesis one was confirmed. All maladaptive core selves and other schemas were positively associated with dissociative experiences. This is in line with theoretical predictions in the sense that when maladaptive core schemas are stable and are activated, it is expected that individuals engage in defensive processes, such as dissociation, which may foster dissociate experiences such as depersonalization, derealization, and amnesia (Bortolon et al., 2017; Krause-Utz, 2022). Thus, Khosravi (2020) documented that EMS of defectiveness/shame, vulnerability to harm and illness, and pessimism were correlated with dissociative experiences in patients with borderline personality disorder. In another study, Karatzias et al. (2016) described a positive correlation between dependent EMS and dissociative experiences in adult survivors of interpersonal trauma. Aligned with this evidence, the present study shows that dysfunctional core cognitions about the self and others are associated with dissociative experiences. Therefore, this study supports the claim that individuals with a dysfunctional perspective about themselves and others are prone to feel dissociative experiences, which is aligned with the cognitive theory of psychopathology (Beck et al., 2015; Young et al., 2003).

The second hypothesis received partial confirmation as adaptive core other-schemas did not correlate with any variable (dissociative experiences and symptomatology). A closer inspection of the DES-28 items suggests that most of the items refer to the experience of the self, which means that they are not focused on the experience of the other. In this sense, the adaptive core and other schemas seem irrelevant to this psychological domain regarding self-dissociative experiences. Faustino, 2022a showed that the adaptive core-other schemas do not correlate with psychological distress, which suggests that this domain may be relatively independent from generic distressful states. However, in the same study, it did correlate negatively with all symptomatology and EMS domains. More research is required to explore these results in the adaptive other dimensions. Moreover, the adaptive core self-schemas correlated negatively with dissociative experiences, suggesting that individuals see themselves as valued, respected, and interesting, they are less likely to report using dissociation to cope with symptomatology.

Hypothesis three was supported. Dialectical Core Schemas and dissociative experiences predicted symptomatology as the hierarchical regression analysis demonstrated. Maladaptive core cognitions about the self and others can be regarded as vulnerability (proponent and precipitant) factors for developing psychopathological symptoms due to the distorted thinking and emotionality that emerges from the dysfunctional appraisals of self and other experiences (Beck et al., 1989, 2015). The same can be said regarding dissociative experiences. An individual who manifests steady distractibility, self-absorption, and/or amnesia because of dissociation,/may likely develop symptomatology, such as anxiety, depression, and/or interpersonal sensitivity (Ellickson-Larew et al., 2020; Seligman & Kirmayer, 2008).

Hypothesis four was also supported. Maladaptive and adaptive core self-schemas mediated the relationship between dissociative experiences and symptomatology. This result suggests that individuals may hold contradictory core cognitions about themselves, which impact several psychological variables, such as coping mechanisms (e.g., dissociation) and symptomatology. On one hand, maladaptive core self-schemas facilitate the relationship between dissociative experiences and symptomatology because when these schemata are activated, individuals see themselves as weak, unworthy, and unlovable, which may give them a sense of weakness, fragility, and vulnerability to dissociative experiences (Bortolon et al., 2017; Young et al., 2003). On the other hand, adaptive core self-schemas may impair the relationship between dissociative experiences and symptomatology because when these schemata are activated, individuals see themselves as strong, capable, and valued, meaning they do not need to avoid distressful feelings and emotions about themselves. In this sense, individuals may swing back and forth on a continuum between two dialectical poles with adaptive and maladaptive core cognitions about themselves (Faustino, 2022a, 2022b).

Finally, the fifth hypothesis received partial confirmation, because the adaptive core other schemas did not mediate the relationship between dissociative experiences and symptomatology. However, the mediation with the maladaptive core of other schemas was significant. This suggests a differential weight of the adaptive other schemas dimension on the relationship between dissociative experiences and symptomatology. As stated before, dissociative experiences are concerned with the dimension of the self and not with the dimension of the other. However, because maladaptive core other schemas were significant, maybe the relationships between dysfunctional mental contents transcend the self-other dichotomy (Faustino, 2022a, 2022b). Aligned with this, dissociative experiences as measured by DES-28 may be more in line with intrapsychic processes rather than interpsychic processes. Thus, this is also suggested by the correlational pattern, where most of the DES-28 subscales (apart from the Amnesia subscale) correlated with maladaptive and adaptive core self-schemas and correlated only with maladaptive other schemas. Another explanation may be that adaptive core other schemas underly adaptive views of others. If individuals see others as trustful, supportive, and accepting, they do not need to defend themselves. In this sense, it is expected the adaptive other domains do not have a significant effect on the relationship between dissociative experiences and symptomatology. However, more research is required to explore this aspect.

### Clinical Implications

The results of the present study may have some clinical implications. DCS may help clinicians and researchers to better understand how individuals experience at the same time opposite cognitions about the self and others and how they impact dissociative experiences. Therefore, case conceptualization may be enhanced by the direct exploration of not only the maladaptive schemas but also their counterpart. Another implication is to explore how individuals disregard their adaptive schemas and how these processes may have a dysfunctional impact on mental health. Processes such as maladaptive coping and lack of behavioral skills (Young et al., 2003), cognitive distortions (Beck et al., 2015), defense mechanisms (Cramer, 2000), psychological inflexibility (Hayes et al., 1999) and emotional processing difficulties (Elliott & Greenberg, 2016), which are associated with EMS (Faustino & Vasco, 2020c, 2020b; Walburg & Chiaramello, 2015) may play a role on how individuals disregard they adaptive views of the self. However, this needs further empirical exploration. Interventions may be focused explicitly on weakening maladaptive and strengthening adaptive schemas. For instance, traumatized individuals will always have a core schema of abused or humiliated self because they effectively experienced humiliation. In this sense, the maladaptive schema is always there but can be weakened by strengthening an adaptive one. Also, individuals with personality disorders may hold deeply rooted core beliefs (Beck et al., 2015), which are extremely hard to change. Therefore, strengthening adaptive schemas that are different from the maladaptive ones may help individuals to better deal with themselves and others. Thus, previous research on early positive schemas suggested that they are negatively associated with symptomatology (Louis et al., 2018; Paetsch et al., 2022; Videler et al., 2020), which is suggestive that they can be strengthened with the objective of reducing symptoms severity. These issues may also be shared with patients to develop a common clinical objective aligned with transtheoretical principles of change and empirically supported tasks, such as imagery reprocessing and chair work.

### Limitations and Future Directions

The findings need to be interpreted within the context of several limitations. Constructs were assessed via self-report measures which may be subject to biases. In addition, self-report instruments may reflect participants’ subjective thoughts and feelings during the assessment, which may not be the same as what they experience in their lives. Given the cross-sectional nature of the current study, we were not able to examine temporal causality. Dissociation can be both a reaction to maladaptive schema activation and, in turn, a schema-perpetuating experience (Lysenko et al., 2018). The distress, difficulties with functioning, and perceived loss of control that accompanies dissociation may reinforce maladaptive schemas (e.g., “I am weak”). Recurrent dissociative experiences can therefore perpetuate a maladaptive sense of self which in turn increases one’s vulnerability to mental health problems (Spiegel et al., 2011). Longitudinal investigations are needed to explore the potential bi-directional nature of the associations between dissociation, dialectical core schemas, and mental health. Our sample had more women than men which may limit the generalizability of the findings to men. Future research could explore these relationships in a gender-balanced sample. Relationships should be explored with clinical samples and with other trauma-related variables, such as early trauma and PTSD symptomatology.

## Conclusions

Maladaptive and adaptive core cognitions about the self are associated with dissociative experiences and symptomatology in a non-clinical sample. These findings have contributed to identifying the mechanisms linking dissociative experiences to mental illness. Case conceptualization and clinical decision-making can be enhanced by considering the complex relationships between dialectical core schemas, dissociation, and symptomatology. Psychological schemas implicated in dissociative experiences and mental disorders are likely to be important treatment targets.
